# Differential effects of specific cathepsin S inhibition in biocompartments from patients with primary Sjögren syndrome

**DOI:** 10.1186/s13075-019-1955-2

**Published:** 2019-07-18

**Authors:** Patrick Hargreaves, Douglas Daoudlarian, Michel Theron, Fabrice A. Kolb, Marianne Manchester Young, Bernhard Reis, Andre Tiaden, Bettina Bannert, Diego Kyburz, Tobias Manigold

**Affiliations:** 1 Department of Rheumatology, University Hospital Basel, Basel, Switzerland; 2 Roche Pharma Research and Early Development, Roche Innovation Center Basel, F. Hoffmann-La Roche, Basel, Switzerland

**Keywords:** Primary Sjögren syndrome, Cathepsin S, Tear fluid, T cell responses, RO5459072

## Abstract

**Objective:**

Primary Sjögren syndrome (pSS) is characterized by T and B cell infiltration of exocrine glands. The cysteine protease cathepsin S (CatS) is crucially involved in MHCII processing and T cell stimulation, and elevated levels have been found in patients with RA, psoriasis and pSS. However, little is known about the functional characteristics and mechanisms of SS-A- and SS-B-specific T cells in pSS patients. We herein investigated the inhibition of CatS activity in different biocompartments of pSS patients including antigen-specific T cell responses.

**Methods:**

Ex vivo CatS activity was assessed in tears, plasma and saliva of 15 pSS patients and 13 healthy controls (HC) and in the presence or absence of the specific CatS inhibitor RO5459072. In addition, antigen (SS-A (60kD), SS-B, influenza H_3_N_2_, tetanus toxoid and SEB)-specific T cell responses were examined using ex vivo IFN-γ/IL-17 Dual ELISPOT and Bromdesoxyuridin (BrdU) proliferation assays in the presence or absence of RO5459072. Supernatants were analysed for IL-1β, IL-6, IL-10, TNF-α, IL-21, IL-22 and IL-23, using conventional ELISA.

**Results:**

CatS activity was significantly elevated in tear fluid, but not other biocompartments, was inversely associated with exocrinic function in pSS patients and could significantly be suppressed by RO5459072. Moreover, CatS inhibition by RO5459072 led to strong and dose-dependent suppression of SS-A/SS-B-specific T cell effector functions and cytokine secretion by CD14^+^ monocytes. However, RO5459072 was incapable of suppressing SS-A/SS-B-induced secretion of cytokines in CD14^+^ monocytes when T cells were absent, confirming a CatS/MHCII-mediated mechanism of suppression.

**Conclusion:**

CatS activity in tear fluid seems to be a relevant biomarker for pSS disease activity. Conversely, CatS inhibition diminishes T cell and associated monokine responses towards relevant autoantigens in pSS. Thus, CatS inhibition may represent a promising novel treatment strategy in pSS.

**Electronic supplementary material:**

The online version of this article (10.1186/s13075-019-1955-2) contains supplementary material, which is available to authorized users.

## Background

Sjögren syndrome is the second most common autoimmune disease in Western countries and has a prevalence of up to 2.7% in Europe [1]. The disease primarily affects exocrine glands leading to their destruction, diminished function and classical dryness of mucosae. In addition, systemic and possibly life-threatening manifestations include encephalitis, vasculitis, lymphocytic pneumonitis, arthritis and lymphoma [2].

As for the pathogenesis, it is believed that follicular T helper cells and B cells enter the glands leading to a lymph-node-like follicular organization. Antigen-presenting cells (APC), including B cells, foster T cell activation and B cell differentiation towards anti-SS-A/SS-B-producing plasma cells [2]. Antibodies are considered to further enhance inflammation by recognizing SS-A and SS-B antigens on the surface of damaged epithelial cells. Consistently, the saliva of pSS patients frequently shows a higher prevalence of anti-SS-A/SS-B antibodies when compared to patients with SLE, RA or healthy controls [3]. Also, the presence of anti-SS-A represents a major pillar of the diagnosis of Sjögren syndrome [4], indicating their specificity for the disease.

Importantly, immunohistochemistry studies showed predominantly Th1 cytokines, perforin and monocyte-derived IL-12 and IL-18 being present in the salivary glands of pSS patients [5], although the Th1/Th2 cytokine ratio may shift throughout the disease [6]. Also, the development of lymphoma seems to correlate with Th1 stimulation, as INF-γ transcripts were higher in patients developing lymphoma [7]. An increasing number of reports from murine models suggest that Th17 cells are key players in the development of pSS. However, evidence for a role of Th17 cells is scarce in pSS patients. A recent ex vivo study in pSS patients did not show differences in peripheral or gland-infiltrating IFN-γ-positive T cell numbers when compared to controls. However, using single-cell analysis, pSS patients showed higher numbers of glandular Th17 cells [8]. Furthermore, analysis of the TCR repertoire of salivary gland-infiltrating T cells suggested a common autoantigen in the glandular tissue.

Despite these advances in understanding the role of T cells for the pathophysiology of pSS, there is only one report investigating SS-B-specific T cells in pSS patients by means of thymidine incorporation assays [9], while other patient studies are usually based on bulk analysis of peripheral T cells either ex vivo or following maximal non-specific stimulation.

There is an increasing number of reports suggesting the involvement of cathepsins (Cat) in autoimmunity. For instance, CatL knock-out mice show decreased susceptibility to collagen-induced arthritis [10]. Likewise, CatS (and L) are significantly elevated in synovial fluid of patients with RA-related knee synovitis and correlate with DAS28, CRP and serum MMP3 levels [11]. Of note, in models of psoriasis, an inflammatory disease of epithelial cells, CatS was found to be the principal protease responsible for IL-36γ cleavage and activation [12]. Of note, in a previous report, IL-36 expression was significantly higher in minor salivary glands of patients with pSS when compared to healthy controls, whereas IL-36R expression was comparable [13].

CatS belongs to the group of cysteine cathepsins and is almost exclusively expressed by antigen-presenting cells (APCs). CatS cleaves the invariant chain (li)-derived propeptide Lip10 (p10) eventually leaving the CLIP (class II-associated invariant chain peptide) on the MHC. As only CLIP-loaded MHCII molecules can be loaded with exogenous peptide sequences within the phagolysosome, cleavage activity of CatS is crucial for antigen presentation to CD4 T cells. Thus, it is conceivable that elevated CatS activity may lead to elevated MHCII expression and autoimmunity, whereas inhibition of CatS may represent a novel approach to suppress MHCII-mediated T cell responses in autoimmunity. As p10 cleavage in APCs is exclusively exerted by CatS, intracellular p10 accumulation represents a valid biomarker for CatS suppression.

In line with this concept, it recently has been shown that CatS activity in tears of primary and secondary Sjögren syndrome patients is higher than in patients with non-Sjögren’s autoimmune diseases or healthy controls [14, 15]. Conversely, in a murine model of Sjögren’s syndrome, the CatS inhibitor Clik60 was capable of profoundly diminishing glandular inflammation and improving salivary flux [16]. Given the above, CatS inhibition seems a plausible therapeutic approach in patients with primary Sjögren’s syndrome. RO5459072 is a potent and highly specific CatS inhibitor developed by Hoffmann-La Roche, Basel. The pharmacodynamic properties of RO5459072 have been thoroughly described by Theron et al. [17]. Specifically, it mediates robust dose-dependent p10 accumulation and downregulation of MHCII of up to 40% in B cells, but not monocytes, both in vitro and in vivo. Consistently, a significant inverse correlation between p10 accumulation and MHCII downregulation was observed. However, to date, it is unknown whether the reduction of MHCII expression leads to relevant suppression of CD4 T cell responses. This is the first report on the effects of RO5459072 in different biocompartments, including T cells, of patients with pSS and healthy controls.

## Methods

### Subjects

A total of 15 patients with primary Sjögren syndrome (pSS) and 13 healthy controls (HC) were enrolled using informed consent forms approved by the local Ethics committee. Diagnosis of pSS was made according to 2016 ACR/EULAR criteria, consisting of a minimum score of four points, derived from salivary gland histology, anti-SS-A positivity, ocular staining score, Schirmer’s test and unstimulated whole saliva flow rate. Clinical activity of pSS patients was determined by ESSDAI scores, ranging from 0 to 34, and included naïve and treated subjects. Characteristics of patients are summarized in Table 1.

**Table 1:** Baseline characteristics of primary Sjögren syndrome (pSS) patients enrolled

Patient	Sex	Age	Disease duration (y, mo)	Anti-SS-A	Anti-SS-B	Biopsy	Therapies	Manifestations	CRP/BSR	ESSDAI score
pSS 1	f	50	7mo	181	0.1	Neg	Artificial tears	Ocular sicca	Normal	0
pSS 2	f	43	11y 11mo	240	75	Pos	None	Ocular, oral and vaginal sicca, glandular swelling	Normal	2
pSS 3	f	76	36y 1mo	240	ND	ND	Artificial saliva	Ocular and oral sicca	Normal	0
pSS 4	f	48	11y 1mo	240	0.1	ND	CFZ-533 *, hydroxychloroquine	Ocular and oral sicca	ND	17
pSS 5	f	65	Unknown	131	3.2	ND	ND	ND	ND	ND
pSS 6	f	50	2y 2mo	240	5	Pos	None	Ocular sicca	Normal	0
pSS 7	m	40	16y 2mo	240	ND	Pos	Prednisolone p.o. and i.v., artificial tears	Ocular and oral sicca haematological manif., vasculitis, glandular swelling, pneumonitis	Elevated ESR & Elevated CRP	34
pSS 8	f	60	6mo	240	320	ND	None	Ocular and oral sicca	Elevated CRP	15
pSS 9	f	60	1mo	240	5	Pos	Artificial tears	Ocular and oral sicca	Normal	5
pSS 10	f	30	7mo	5	5	Pos	None	Ocular and oral sicca	Normal	0
pSS 11	m	60	1y 6mo	240	6	Pos	None	Ocular and oral sicca	Normal	1
pSS 12	f	53	3y 1mo	240	320	Pos	Methotrexate, hydroxychloroquine, artificial saliva	Ocular and oral sicca	Normal	6
pSS 13	f	51	9mo	20	ND	ND	Hydroxychloroquine, artificial tears, artificial saliva	Sicca	Normal	4
pSS 14	f	64	37y 6mo	240	298	Pos	Artificial saliva	Ocular and oral sicca	Normal	3
pSS 15	f	53	10mo	240	ND	Pos	Methotrexate, hydroxychloroquine, artificial saliva	Ocular, oral and vaginal sicca	Normal	8

### Preparation of biofluids

Tears were collected as previously described [15]; thereafter, the derived fluid was pooled, aliquoted, and stored at − 80 °C until analysis (mean freezing period 23 days). Plasma was collected after centrifugation of whole blood and stored at − 20 °C until analysis within 3 months. Unstimulated saliva was collected every minute over a period of 5 min. Likewise, stimulated saliva was collected after chewing a piece of paraffin during a 5-min period. Saliva samples were centrifuged, and supernatants stored at − 80 °C until use within 3 months.

### Collection and preparation of PBMC

Peripheral blood was collected in heparinized tubes, and mononuclear cells were isolated using Leucosep tubes (Greiner Bio-One, St. Gallen, Switzerland) and Ficoll-Paque density gradient centrifugation as described by the manufacturer. Peripheral blood mononuclear cells (PBMC) thereafter were applied freshly to ELISPOT and proliferation assays as indicated below.

*Fluorometric assessment of p10* was carried out as described before [17]. Briefly, PBMC were pelleted, fixed and permeabilized before being stained with unlabelled rabbit anti-p10 antibody (Hoffmann-LaRoche). PE-conjugated, goat anti-rabbit IgG antibody (SouthernBiotech, Birmingham, AL, USA) was then added to the cells. Finally, the cells were stained with PerCP-Cy5.5-conjugated anti-CD45 (BioLegend, London, UK), BV421-conjugated anti-CD3 (BioLegend, London, UK), APC-conjugated anti-CD14 (Beckman Coulter, Nyon, Switzerland) and Alexa Fluor 488-conjugated anti-CD20 (BD, Allschwill, Switzerland) antibodies. FACS analysis included gating on CD45-positive events to select leukocytes, followed by doublet-excluding gates on forward scatter area v. height and side scatter area v. height, followed by gates defining CD3-positive/-negative and CD20-positive/-negative subsets. A CD14-positive subset was defined within the CD3-negative/CD20-negative gate. Flow cytometry data analysis software FlowJo (Ashland, OR, USA) was used to further derive median fluorescence intensity (MFI) values for Lip10 (p10, MFI) of B cells and T cells in all samples and of monocytes in human samples. Additionally, a p10 stain index for B cells was calculated for each sample by dividing the p10 MFI value of the B cell population by that of the T cell population. A p10 stain index for monocytes was also calculated for each human sample by dividing the p10 MFI value of the monocyte population by that of the T cell population.

### Antigens

Soluble substance A (SS-A (60 kDa); MyBioscource Inc., San Diego, USA), soluble substance B (SS-B, Fitzgerald Industries Intl., Acton, USA), tetanus toxoid (TT, Merck-Millipore, Darmstadt, Germany), influenza hemagglutinin 3 neuroaminidase 2 (H_3_N_2_, Sino Biologicals, Beijing, China), and *Staphylococcus aureus* enterotoxin B (SEB, Sigma-Aldrich, Darmstadt, Germany). Ultrapure Lipopolysaccharide (LPS, *E. coli* 0111:B4, InvivoGen, San Diego, USA) was used at 100 ng/ml. All recombinant antigens were reconstituted and stored according to the manufacturer’s recommendations and aliquoted for single use to avoid freeze-thaw cycles.

### ELISPOT assay

Numbers of antigen-specific IFN-γ and IL-17 spot forming units (SFU) were assessed using the dual-colour ELISPOT assay (Cellular Technologies Ltd., Bonn, Germany). Triplicates of 2 × 10^5^ PBMC/well were incubated for 48 h for each condition.

### BrdU/proliferation assay

Triplicates of 5 × 10^4^ PBMC/well for each condition were placed in U-shaped bottom 96-well-plates for 72 h. BrdU incorporation was assessed as indicated by the manufacturer (Roche Diagnostics GmbH, Mannheim, Germany). The stimulation index (SI) was calculated by dividing values of antigen conditions by values of untreated cells.

### Cathepsin S and cathepsin L activity assays

CatS and CatL activity in the presence and absence of RO5459072 was determined using the SensoLyte® 440 Fluorimetric Cathepsin S and Cathepsin L Assay Kit (Anaspec, CA, USA) according to the manufacturer’s instructions, respectively.

### Cathepsin S and cathepsin L concentration assays

CatS and CatL concentrations were measured in tear, plasma, unstimulated saliva and stimulated saliva samples using the Human Total Cathepsin S or Cathepsin L DuoSet ELISA (RnD Systems Inc., Minneapolis, USA) according to the manufacturer’s instructions, respectively.

### ELISA

IL-1β, IL-6, IL-10, IL-12p70, IL-23 and TNF-α (eBioscience, Science Center Dr. CA, USA); IL-21 (Mabtech, 28 Nacka Strand, Sweden); and IL-22 (Thermofischer, Campus Vienna, 1030 Vienna Austria) were used for the assessment of the supernatants of Elispot experiments containing 2 × 10^5^ cells/well following 48-h incubation with the respective antigens. ELISA was performed according to the manufacturer’s instructions using 25 uL/well of sample per well of a 384-well plate. IL-1β and TNF-α were analysed using 1:10 dilutions for all subjects except for pSS 1 and pSS 3, whereby IL-1β was analysed using a 1:4 dilution. All other cytokines were analysed using 1:2 dilutions.

### CD14 cell purification

Enrichment of CD14^+^ cells from PBMC was performed using CD14 MicroBeads (Milteny Biotec) according to the manufacturer’s instructions. MACS MS Columns were washed, and both CD14^−^ cells and CD14^+^ cells were used for subsequent experiments, as indicated in the legend of Fig. 5.

### Statistical analysis

Results for all tests comparing pSS and HC groups used the Mann-Whitney *U* test (two-tailed, *p* < 0.05). Association between parameters has been evaluated using the Spearman rank correlation. Results were analysed using statistical methods as indicated (see legends). GraphPad Software version 8.1 (San Diego, USA) was used for all statistical analyses.

## Results

### Functional characteristics and CatS in different biocompartments

**Fig. 1:**
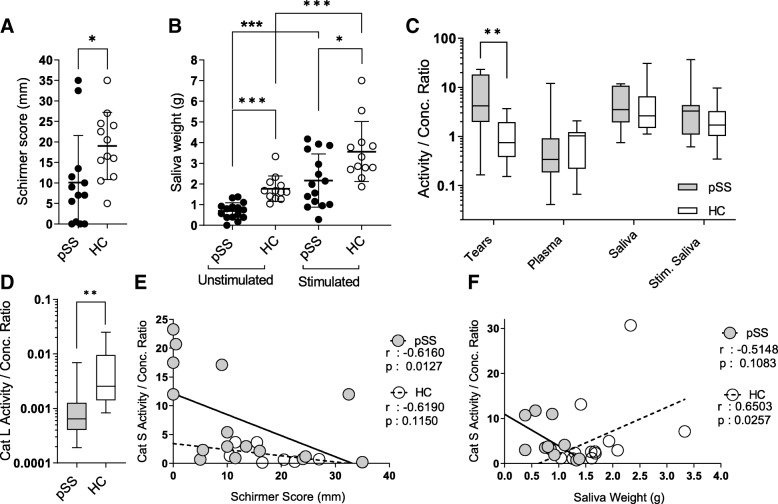
Functional characteristics and cathepsin S in different biocompartments. Primary Sjögren syndrome (pSS) patients showed significantly reduced ocular tear production (**a**) and reduced spontaneous or stimulated salivary flow, when compared to healthy controls (HC) (**b**). CatS (activity/conc) ratios were significantly elevated for tears of pSS when compared to HC, whereas no significant increase could be observed for the other biocompartments (**c**). By contrast, ratios for CatL, an endopeptidase involved in collagen and elastin degradation during necrotic and tumorous processes, were significantly decreased in tears of pSS as when compared to HC (**d**) (Mann-Whitney *U* test, *p < 0.05, **p < 0.01, ***p < 0.001, ****p < 0.0001). An inverse association between tear (**e**) and saliva **(f)** production and CatS ratios in pSS individuals is suggesting a CatS-dependent inflammatory process as causative for the decreased exocrine function (Spearman’s rank correlation, one-tailed *t* test, respectively)

A total of 15 pSS and 13 HC individuals were enrolled. The patient characteristics are summarized in Table 1. As expected, pSS showed significantly reduced ocular tear production (Fig. 1a) and reduced spontaneous or stimulated salivary flow, when compared to healthy controls (Fig. 1b). Of note, stimulation by chewing gum significantly improved salivary flow in pSS patients and healthy controls, respectively.

Next, CatS activity in different biocompartments of pSS and HC individuals were normalized by CatS concentrations. CatS ratios were significantly elevated for tears of pSS when compared to HC individuals, whereas no significant differences could be observed for the other biocompartments (Fig. 1c). By contrast, ratios for CatL, an endopeptidase involved in collagen and elastin degradation during necrotic and tumorous processes, were significantly decreased in tears of pSS when compared to HC (Fig. 1d). Our hypothesis, that elevated CatS activity is associated with decreased glandular function, was supported by an inverse association between tear (Fig. 1e) and saliva (Fig. 1f) production and CatS ratios in tears of pSS individuals but not healthy controls.

### Effects of RO5459072 on ex vivo CatS activity

As recently shown, RO5459072 leads to dose-dependent p10 accumulation in B cells [17]. We could reproduce these data and observed median IC 50 values of 11.53 nM in pSS and 25.95 nM in HC individuals (Additional file 1: Figure S1A). In addition, 80 μM of RO5459072 led to significantly decreased CatS ratios in tear fluid but not in other biocompartments of pSS (Additional file 1: Figure S1B).

### Effects of RO5459072 on ex vivo functions of specific T cells

**Fig. 2:**
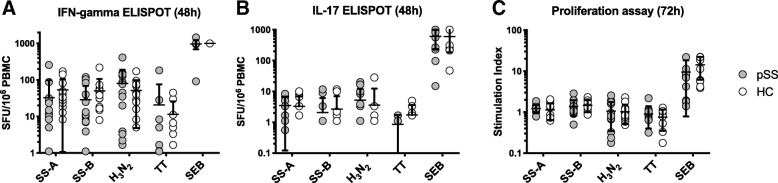
T cell responses towards SS-A/SS-B in pSS and HC. Antigen-specific T cell responses in pSS patients (grey circles) and HC (open circles) were examined in the absence of RO5459072 using IFN-γ/IL-17 dual ELISPOT (**a**, **b**) and proliferation assays (**c**). Surprisingly, there were no overall differences between pSS and HC with respect to frequencies of IFN-γ- and IL-17-secreting and proliferating T cells. Of note, whereas up to 250/10^6^ PBMC-specific IFN-γ-secreting cells could be observed in some individuals, no relevant numbers of SS-A/SS-B-specific Th17 cells could be detected

T cell responses in pSS patients and healthy controls were studied using IFN-γ/IL-17 dual ELISPOT (Fig. 2a and b) and proliferation assays (Fig. 2c). Surprisingly, there was a high degree of variability in T cell responses towards SS-A and SS-B in both groups. The responses ranged from basically absent to up to 258 SFU/10^6^ PBMC for IFN-γ and 2.838 SI, respectively. No significant differences between patients and healthy controls were detected. Of note, no relevant numbers of SS-A/SS-B-specific Th17 cells were detected in either patients or healthy controls.

**Fig. 3:**
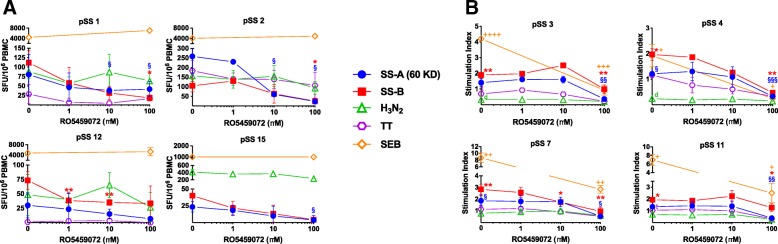
Dose-dependent suppression of SS-A/SS-B-specific T cell responses by RO5459072. Of note, some pSS patients showed strong IFN-γ secretion (**a**) but no relevant proliferation towards SS-A/SS-B, whereas others were IFN-γ negative but showed specific proliferation (**b**). RO5459072 significantly diminished SS-A- and SS-B-specific T cell responses but also was capable of massively reducing SEB-induced proliferation of T cells (one-tailed *t* test * or § *p* < 0.05, ** or §§ or ++ *p* < 0.01, §§§ or +++ *p* < 0.001; ++++ *p* ≤ 0.0001), suggesting a mode of action independent of MHCII regulation

We furthermore investigated the effect of CatS inhibition in eight pSS patients with strong T cell responses (“T cell responders”, defined as ≥ 10 SFU/10^6^ PBMC in ELISPOT (Fig. 3a) or significant responses in proliferation assay (Fig. 3b) when compared to unstimulated control) in the absence or presence of RO5459072. Interestingly, pSS individuals with high frequencies of SS-A- or SS-B-specific IFN-γ-producing T cells (pSS1, pSS2, pSS10, pSS13) did not show relevant proliferative responses, whereas others (pSS3, pSS4, pSS6, pSS9) had low IFN-γ responses but showed significant specific proliferation. RO5459072 significantly diminished specific T cell responses in a dose-dependent manner. The suppressive effect was somewhat more pronounced for SS-A/SS-B-specific T cells than for tetanus- or influenza-specific responses. Similar effects were seen in samples from HC with considerable ex vivo T cell responses to SS-A/SS-B (Additional file 2: Figure S2).

Of note, RO5459072 was also capable of massively reducing SEB-stimulated T cell proliferation, whereas SEB-induced IFN-γ secretion remained largely unaffected.

### Effects of CatS inhibition on SS-A- and SS-B-induced monokine production

We next studied seven cytokines (IL-1β, IL-6, IL-10, TNF-α, IL-21, IL-22 and IL-23) in 48-h supernatants of ELISPOT assays of seven pSS patients with strong T cell responses (“T cell responders”), three pSS patients without significant SS-A/SS-B-specific responses in ELISPOT or proliferation assay, respectively (“T cell non-responders”), and four HC without SS-A/SS-B-specific T cell responses.

**Fig. 4:**
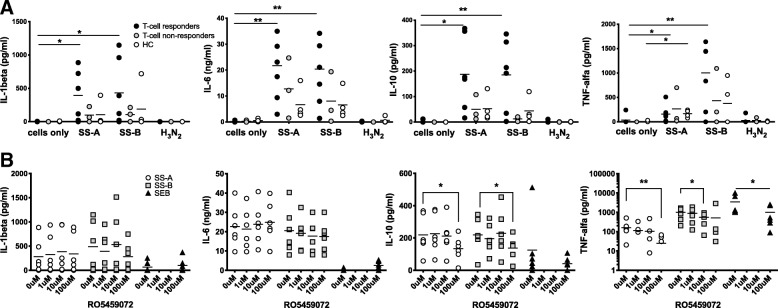
Upper panel: SS-A and SS-B are potent monokine inducers. Both proteins induce IL-1β, IL-6, IL-10 and TNF-α in pSS patients with high T cell responses (responders (*n* = 7), see Fig. 4) but not in non-responders (*n* = 3) or HC (*n* = 4, except TNF-α for SS-A), (two-tailed *t* test *p < 0.05, **p < 0.01). Lower panel: RO5459072 suppresses monokines induced by SS-A and SS-B in pSS patients with strong cognate T cell responses (*n* = 6, see Fig. 4, one-tailed *t* test *p < 0.05, **p < 0.01)

Interestingly, recombinant SS-A and SS-B were strong stimulators of IL-1β, IL-6, IL-10, and TNF-α in most T cell responders, whereas H_3_N_2_ was not inductive (Fig. 4a). T cell responders showed significantly higher cytokine levels than T cell non-responders. As for IL-21, IL-22 and IL-23, no relevant levels were observed following SS-A/SS-B incubation in either pSS patients or healthy controls (data not shown). Despite a trend towards cytokine induction in the pSS T cell non-responders and HC, significance levels were not reached, except for TNF-α following SS-A incubation in HC. Importantly, co-incubation with 25 μg/ml polymyxin B did not abrogate SS-A- or SS-B-induced TNF-α secretion (data not shown), indicating the absence of relevant endotoxin contamination of SS-A/SS-B proteins. Of note, in PBMC cultures (supernatants derived from 48 h ELISPOT assays), RO5459072 significantly diminished TNF-α and IL-10 levels induced by SS-A/SS-B, whereas no suppression was observed for IL-6 and IL-1β, respectively (Fig. 4b). As for IL-6, it is noteworthy that SEB-induced IL-6 levels were significantly reduced by RO5459072, whereas those induced by SS-A and SS-B were not.

### CD14^+^ cells are the principal source of SS-B-induced cytokines

**Fig. 5:**
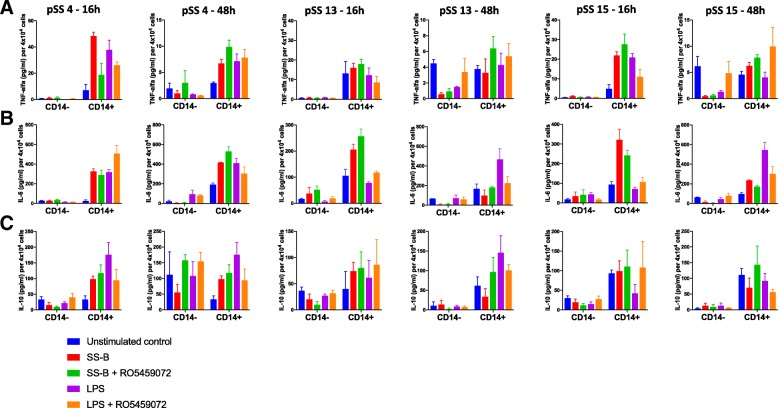
CD14^+^ cells were isolated from frozen PBMC of three pSS individuals, as described in the “Methods” section. Either 2 × 10^5^ CD14^−^ cells/well or 4 × 10^4^ CD14^+^ cells/well were incubated with indicated proteins for 16 h and 48 h, in presence or absence of 100 μM RO5459072, respectively. Subsequently, supernatants were analysed by ELISA for TNF-α **(a)**, IL-6 **(b)** and IL-10 **(c)**. Results were normalized to 4 × 10^4^ cells/well. Values reflect mean of triplicates + standard error of the mean

In a next step, we sought to investigate the cellular source of SS-A/SS-B-induced cytokine secretion. As SS-B seemed to exert higher TNF-α levels (Fig. 4a), we restricted subsequent experiments to this protein. As shown in Fig. 5, bead-enriched CD14^+^ monocytes were\ the main source for SS-B-induced secretion of TNF-α and IL-6 after 16 h of stimulation, whereas the effects were reduced after 48 h of stimulation: the stimulatory effect was most consistent for IL-6 and less pronounced for IL-10 for both time points. Coincubation with 100 μM of RO5459072 was incapable of suppressing monokine production to a significant extent (using paired *t* test). Of note, SS-B induced cytokines reached levels similar to or higher levels than those induced by 100 ng/ml LPS. By contrast, CD14-depleted PBMC did not show levels above unstimulated control conditions for most conditions, in response neither to SS-B nor to LPS, thus confirming effective CD14-depletion.

## Discussion

In line with a previous report in pSS, we found elevated CatS activity in tear fluid and an inverse association of CatS (activity/concentration) ratios with exocrine glandular functions [14, 15]. This finding and data from a longitudinal model in NOD mice [18] support the notion of CatS-mediated inflammation in pSS and suggests that CatS in tears may indeed serve as a relevant marker of disease state. Importantly, the stability of CatS in tear fluid was not significantly influenced by time of storage (Additional file 3: Figure S3). RO5459072 also diminished CatS activity in tear fluid of pSS, whereas no significant effect could be observed in other biocompartments. While in plasma this may have been due to low absolute levels of CatS ratios, it is less conceivable for saliva, where CatS ratios were comparable to those of tears. A possible explanation may be the presence of additional saliva enzymes capable of processing the CatS substrate used in our functional assay, thus rendering CatS inhibition by RO5459072 less effective. Interestingly, we could not find significant correlations between disease duration and CatS or CatL expression. This may however also be owed to the low number of pSS patients enrolled in our study. Another limitation of our study is that we did not include patients with other rheumatic diseases and secondary Sjögrens or patients with non-immune sicca symptoms. We thus cannot conclude that our findings are specific for primary Sjögren Syndrome. However, our data in pSS patients coincide with the previous work of Hamm-Alvarez et al. and Edman et al. [14, 15], in which patients with secondary SS, but not non-immune sicca patients, showed increased CatS levels in tears similar to patients with pSS. Conclusively, the inverse correlation between CatS ratios and exocrinic gland function is in line with a model, where CatS/MHCII-dependent CD4 T cell responses lead to the destruction of the gland, which in turn results into decreased tear and saliva production.

Furthermore, to our knowledge, there is only one report on autoantigen-specific T cells in patients with Sjögren’s disease and healthy controls. This report estimated the SS-B-specific precursor T cell frequency at 10^−5^ to 2 × 10^−5^ using thymidine proliferation assays [9]. We found up to 250 SS-A/SS-B-specific IFN-γ-producing T cells per 10^6^ PBMC, which might be explained by a vastly higher sensitivity of ELISPOT assays as compared to proliferation assays. It is widely known that frequencies of auto-antigen-specific T cells can largely overlap in healthy subjects and patients with autoimmune disease, such as type 1 diabetes, Graves’ disease and rheumatoid arthritis. Interestingly, not only frequencies but also functions of specific T cell subsets were comparable between patients and healthy subjects [19–21]. Similarly, we herein confirmed the findings by Helsloot et al., showing that healthy controls also show readily detectable frequencies of SS-antigen-specific T cells [22]. In our study, there were no significant differences in T cell frequencies between and pSS individuals, reaching as many as 193 SFU/10^6^ PBMC in healthy controls. However, as only pSS patients were positive for anti-SS-A/SS-B antibodies, our data suggest that additional factors beyond SS-A/SS-B-specific T cells are responsible for seroconversion and eventually for disease development. Importantly, the phenotype and functions of peripheral T cells may not be identical to those within the affected glandular tissues. Whether, e.g., a disbalance in tolerogenic dendritic cells, regulatory T cells or other factors favouring B cell maturation eventually are determinants for seroconversion and, eventually, disease development remains speculative. However, considering that anti-SS-A/SS-B antibodies can be detected in the saliva of pSS patients [23] and the lymph-node-like follicular structures classically observed in glandular tissue of pSS patients makes it likely that the determinant factors have to be sought at the site of inflammation, namely the glandular tissue. In the same context, it is important to note that we could not find evidence for circulating SS-A/SS-B-specific IL-17-secreting cells using highly sensitive Dual ELISPOT. While Th17 cells are discussed as a crucial factor in the pathogenesis of pSS in murine models, ex vivo studies in pSS patients show contradictory results [24]. Again, one explanation is that peripheral T cell subsets are not representative of glandular subsets. However, most recent evidence from genomic approaches in saliva and salivary glands from pSS patients do not show evidence for a prevalent Th17 signature [25, 26]. In addition, discrepancies among previous studies describing relevant numbers of Th17 in pSS patients may derive from the detection of surface markers in some studies, whereas others were based on direct cytokine detection [27, 28]. Also, T cell studies in pSS patients were almost exclusively performed either directly ex vivo or following bulk-stimulation with strong mitogens or PMA/ionomycin, but not SS-A or SS-B proteins.

Following incubation with RO5459072, we herein observed comparable p10 kinetics in B cells as reported before [17, 29]. Notably, in pSS, the mean IC_50_ value was approximately 50% of that in HC, suggesting a higher susceptibility of B cells of pSS patients. This is different from samples of patients with systemic lupus erythematosus where IC_50_ values were slightly higher than in healthy control samples [29]. Since cleavage of p10 to CLIP by CatS is crucial for loading of the MHCII molecule in the phagolysosome and eventually transports to the cell surface, we (data not shown) and others [17] found HLA-DR downregulation following RO5459072 incubation. As previously shown in mice, accumulation of p10 and impairment of MHCII-dependent antigen presentation in the context of CatS deficiency seems to depend on both the class II allele and the type of the exogenous antigen [30]. Specifically, CatS deficiency did not result in accumulation of li fragments I-A^q^ molecules, whereas this was the case with H-2^q^ and H-2^b^ alleles. This indicates allele-dependent li-fragment dissociation and, thus, limited impact of CatS function. This may also explain why RO5459072 resulted in MHCII reduction of only up to 40% in B cells [17]. Likewise, not all exogenous antigens show the same susceptibility to CatS deficiency. In the context of the H-2^q^ allele, CatS deficiency showed a normal presentation of hen egg lysozyme and ovalbumin, whereas presentation of collagen II was inhibited. The latter mechanism also resulted in increased resistance towards collagen-induced arthritis [31]. Given this evidence, it remained unclear whether and to which extent specific CatS inhibition by RO5459072 would translate into impairment of SS-A/SS-B-specific CD4 T cell responses in pSS patients. In our study, RO5459072 massively downregulated specific T cell responses towards SS-A/SS-B, whereas influenza- and TT-specific IFN-γ responses remained largely unaltered. Thus, SS-A/SS-B affinity to a wide range of class II molecules seems to be sufficiently high to render the cognate T cell responses susceptible to CatS inhibition. Whether the lack of suppression of influenza and TT-specific responses is to be attributed to differences in their affinity to the class II alleles cannot be answered by our study. Alternative explanations may include differences in the differentiation status of SS-A/SS-B- versus influenza/TT-specific T cells. These may result in different activation thresholds of specific T cell subsets and, thus, in differential susceptibility to CatS-dependent MHCII presentation. SEB, by contrast, induces maximal T cell stimulation by forming covalent bindings between TCR and MHCII molecules [32] and was added simultaneously with RO5459072 to our T cell cultures. The fact that RO5459072 had little effect on SEB-induced IFN-γ secretion, whereas proliferation was massively abrogated, may indeed indicate different levels of differentiation of the affected T cell subsets. To investigate the phenotype of SS-A/SS-B-specific T cells, we tried using intracellular cytokine staining in flow cytometry on a limited number of frozen samples (data not shown). However, we were unsuccessful to acquire sufficient numbers of cytokine-secreting cells to draw reliable conclusions. New prospective studies using fresh cells will be necessary to systematically investigate the phenotype of SS-A/SS-B-specific T cells.

Interestingly, we also found a strong cytokine-inducing effect of SS-A and SS-B, which could partially (IL-10 and TNF-α) be downregulated by RO5459072. This is in line with observations in a murine model of lupus erythematosus [33] and PBMC derived from lupus patients [29]. However, it remained unclear whether this is a result of direct interaction of RO5459072 on macrophages or depends on the CatS/MHCII modulating mode of action of RO5459072. Of note, Thanei et al. found cytokine-suppressive effects of RO5459072 in M1, but not M2, macrophages and were accompanied by the downregulation of MHCII and the costimulatory factor CD80 [29]. Moreover, there is evidence that CatS split products of li upregulate CCL2 through NFκB and that inhibition of CatS leads to the downregulation of CCL2, IL-6, CXCL2 and CCL5 [33, 34]. Thus, a direct mode of action of RO5459072 seems possible, at least in M1 macrophages. We herein show the SS-A/SS-B-induced monokine secretion in both PBMC and CD14^+^ monocyte cultures. However, suppression of monokines by RO5459072 was only observed in PBMC cultures. Moreover, significantly higher amounts of monokines were observed in T cell responders than T cell non-responders and HC (Fig. 4a). Taken together, this suggests that reduction of monokine secretion upon treatment with RO5459072 is a result of reduced T cell activation due to CatS-dependent MHCII presentation. However, once monocytes differentiate into M1 macrophages, additional direct effects of RO5459072 on macrophages may occur.

Taken together, we herein confirm previous evidence that CatS in tear fluid correlates with disease and inversely correlates with exocrinic functions in Sjögren’s disease. This suggests a CatS/MHCII-dependent destruction of glandular tissue. Moreover, RO5459072 showed reliable CatS inhibitory effects in B cells and tear fluid, which is consistent with evidence from others. We extend previous knowledge by analysing consequences of CatS inhibition downstream of antigen-presenting cells by showing profound and consistent immunosuppressive effects on SS-A/SS-B-specific T cell responses as well as subsequent monokine induction. Future studies will have to show whether these in vitro results translate to in vivo effects in patients with pSS.

## Conclusion

CatS activity in tear fluid seems to be a relevant biomarker for pSS disease activity. Conversely, CatS inhibition diminishes T cell and monocyte functions towards relevant autoantigens and thus represents a promising treatment target. Specific CatS inhibition leads to impaired T cell and monokine responses to relevant autoantigens in pSS patients. This confirms the principal mode of action of RO5459072 that is decreased MHCII/peptide presentation by APCs due to inhibition of CatS. Future studies will have to show whether the concept of CatS inhibition may lead to clinically relevant improvement of glandular function and systemic complications in pSS patients.

## Additional files

Additional file 1: **Figure S1.** Ex vivo effects of RO5459072. RO5459072 led to a dose-dependent p10 accumulation in B cells when compared to monocytes (A). In addition, 80 mM of RO5459072 led to significantly decreased CatS ratios in tear fluid, but not other biocompartments, of pSS after an incubation of 180 min (B, two-tailed *t* test, ** *p* < 0.01). (PPTX 91 kb)

Additional file 2: **Figure S2.** Antigen-specific T cell responses in healthy controls (HC), assessed by IFN-γ/IL-17 Dual ELISPOT assays for 48 h (A) and BrdU proliferation assays for 72 h (B) following stimulation with 5 μg/ml recombinant SS-A (60kD), SS-B, influenza H_3_N_2_ or 2 μg/ml tetanus toxoid or 0.1 mg/ml SEB in the absence or presence of RO5459072 (1–100 μM). For details, see material and methods or legend of Fig. 3. (one tailed *t* test *, §, π, δ or + *p* < 0.05; **, §§ *p* < 0.01, +++, §§§, *** or δδδ *p* < 0.001; ++++ *p* ≤ 0.0001). (PPTX 136 kb)

Additional file 3: **Figure S3.** Stability of CatS activity in tear fluid upon storage at − 80 °C. Specifically, tear fluid was stored at − 80 °C on the day of sample acquisition for 7–8 days until the first measurement. After testing, they were re-stored at − 80 °C over a period of 6 (HC1 and pSS2) and 8 (pSS1) weeks, respectively, until the second measurement. As indicated, CatS activity was stable, with a trend towards higher levels in the second test, but below 20% of variation, which is considered acceptable for bioassays. (PPTX 67 kb)

## Data Availability

The datasets used and/or analysed during the current study are available from the corresponding author on reasonable request. All data generated or analysed during this study are included in this published article [and its Additional files].

## Abbreviations

APC: Antigen-presenting cell
BrdU: Bromdesoxyuridin
CatS: Cathepsin S
H_3_N_2_: Influenza hemagglutinin 3 neuroaminidase 2
HC: Healthy controls
li: Invariant chain
pSS: Primary Sjögren syndrome
SEB: *Staphylococcus aureus* enterotoxin B
SS-A: Soluble substance A
SS-B: Soluble substance B
TT: Tetanus toxoid
