# Supplementary figures and images for: Differential effects of specific cathepsin S inhibition in biocompartments from patients with primary Sjögren syndrome
Source: Arthritis Res Ther. 2019 Jul 18;21:175. doi: 10.1186/s13075-019-1955-2 (PMC6637481; doi:10.1186/s13075-019-1955-2)

## Slide 1
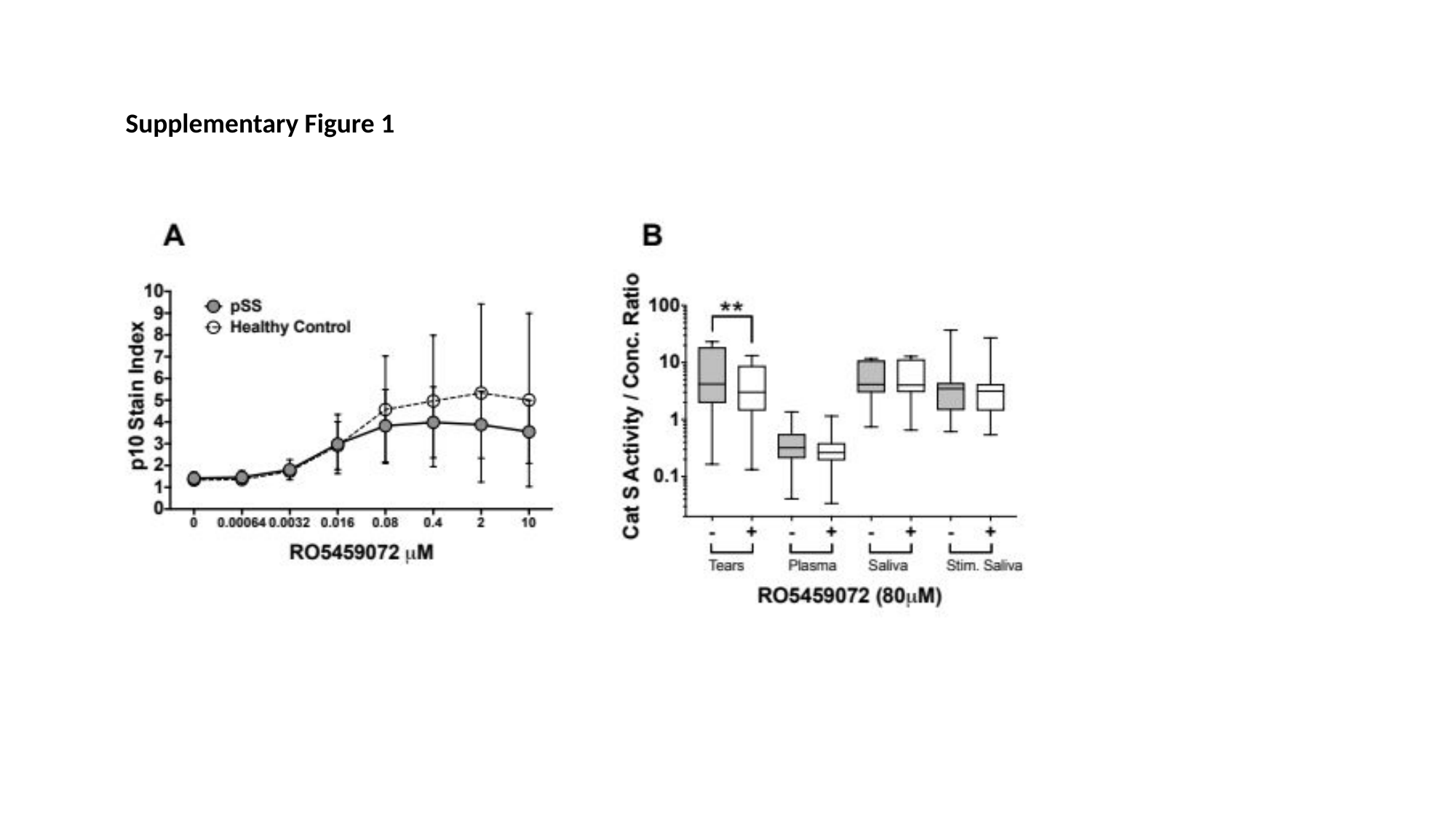

Supplementary Figure 1

Supplement: Supplementary file 1 — Figure S1. Ex vivo effects of RO5459072. RO5459072 led to a dose-dependent p10 accumulation in B cells when compared to monocytes (A). In addition, 80 mM of RO5459072 led to significantly decreased CatS ratios in tear fluid, but not other biocompartments, of pSS after an incubation of 180 min (B, two-tailed t test, ** p < 0.01). (PPTX 91 kb) [file 13075_2019_1955_MOESM1_ESM.pptx]

## Slide 1
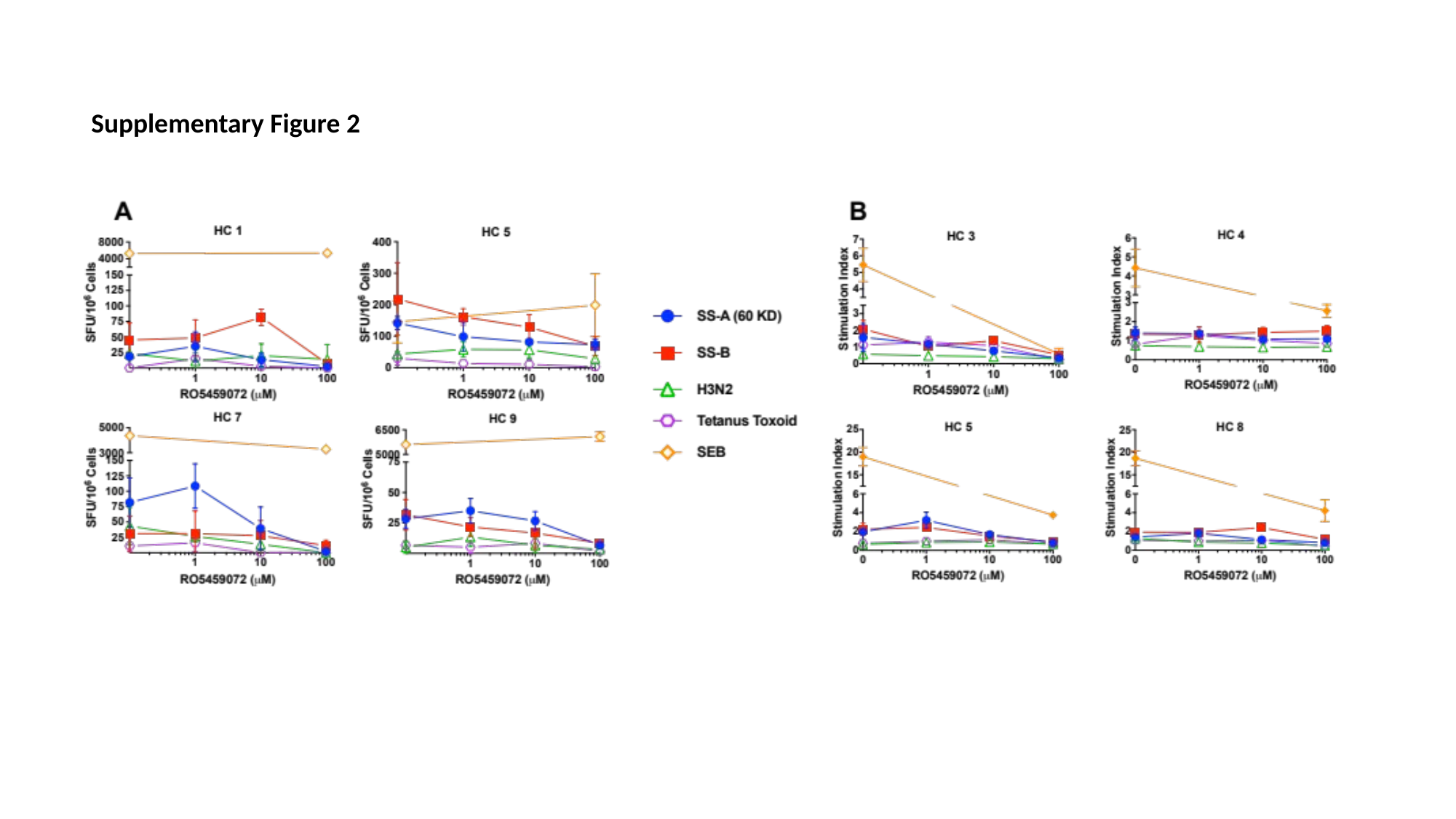

Supplementary Figure 2

Supplement: Supplementary file 2 — Figure S2. Antigen-specific T cell responses in healthy controls (HC), assessed by IFN-γ/IL-17 Dual ELISPOT assays for 48 h (A) and BrdU proliferation assays for 72 h (B) following stimulation with 5 μg/ml recombinant SS-A (60kD), SS-B, influenza H3N2 or 2 μg/ml tetanus toxoid or 0.1 mg/ml SEB in the absence or presence of RO5459072 (1–100 μM). For details, see material and methods or legend of Fig. 3. (one tailed t test *, §, π, δ or + p < 0.05; **, §§ p < 0.01, +++, §§§, *** or δδδ p < 0.001; ++++ p ≤ 0.0001). (PPTX 136 kb) [file 13075_2019_1955_MOESM2_ESM.pptx]

## Slide 1
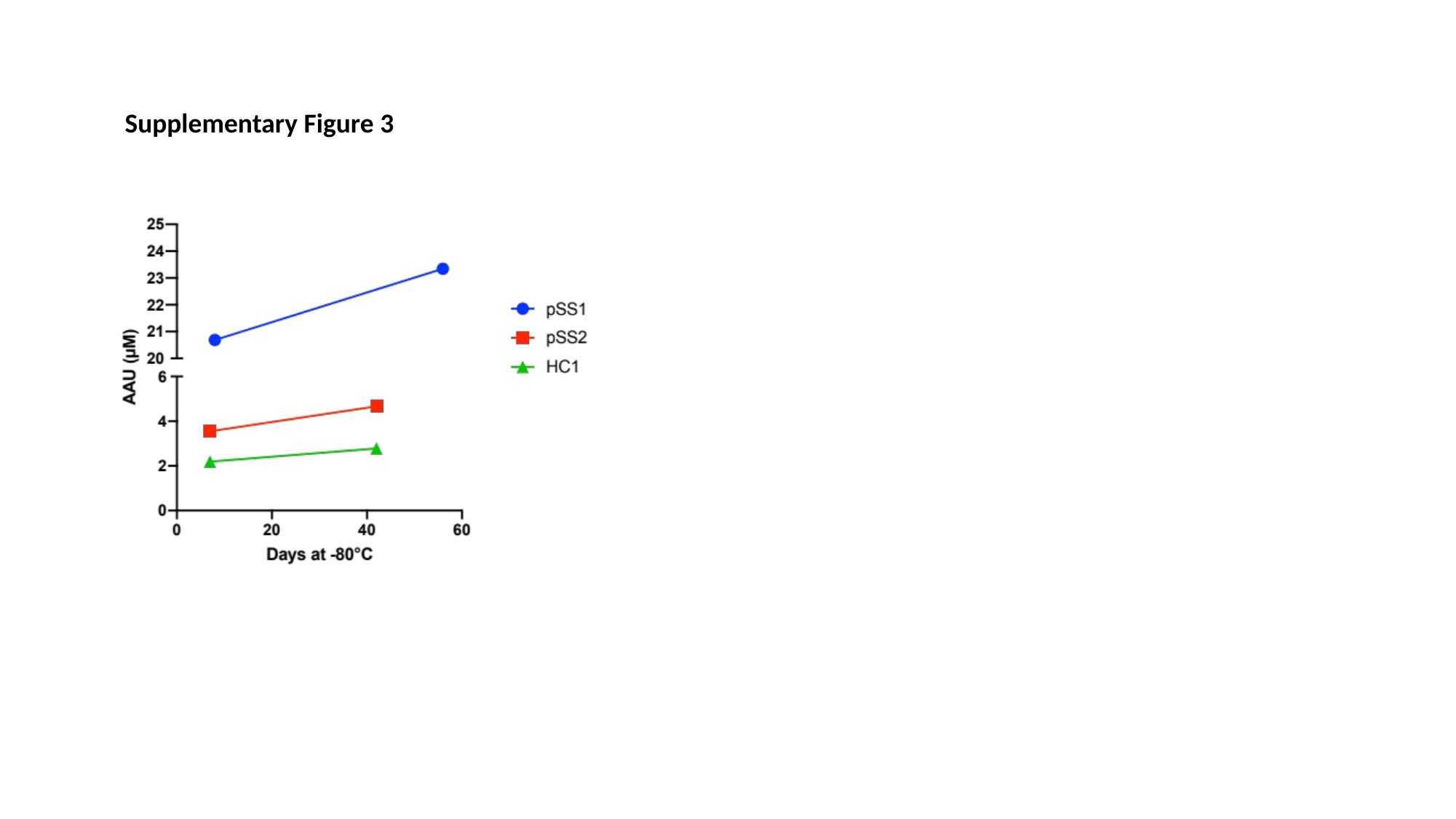

Supplementary Figure 3

Supplement: Supplementary file 3 — Figure S3. Stability of CatS activity in tear fluid upon storage at − 80 °C. Specifically, tear fluid was stored at − 80 °C on the day of sample acquisition for 7–8 days until the first measurement. After testing, they were re-stored at − 80 °C over a period of 6 (HC1 and pSS2) and 8 (pSS1) weeks, respectively, until the second measurement. As indicated, CatS activity was stable, with a trend towards higher levels in the second test, but below 20% of variation, which is considered acceptable for bioassays. (PPTX 67 kb) [file 13075_2019_1955_MOESM3_ESM.pptx]
